# Electrochemically Activated Screen-Printed Carbon Sensor Modified with Anionic Surfactant (aSPCE/SDS) for Simultaneous Determination of Paracetamol, Diclofenac and Tramadol

**DOI:** 10.3390/ma14133581

**Published:** 2021-06-26

**Authors:** Jędrzej Kozak, Katarzyna Tyszczuk-Rotko, Magdalena Wójciak, Ireneusz Sowa

**Affiliations:** 1 Faculty of Chemistry, Institute of Chemical Sciences, Maria Curie-Skłodowska University in Lublin, 20-031 Lublin, Poland; jedrekkozak@onet.pl; 2 Department of Analytical Chemistry, Medical University of Lublin, 20-093 Lublin, Poland; i.sowa@umlub.pl

**Keywords:** electrochemically activated screen-printed carbon electrode modified with sodium dodecyl sulfate, simultaneous determination of paracetamol, diclofenac and tramadol, differential pulse adsorptive stripping voltammetry, river water, human serum and pharmaceutical formulation samples

## Abstract

In this work, an electrochemically activated screen-printed carbon electrode modified with sodium dodecyl sulfate (aSPCE/SDS) was proposed for the simultaneous determination of paracetamol (PA), diclofenac (DF), and tramadol (TR). Changes of surface morphology and electrochemical behaviour of the electrode after the electrochemical activation with H_2_O_2_ and SDS surface modification were studied by scanning electron microscopy (SEM), cyclic voltammetry (CV), and electrochemical impedance spectroscopy (EIS). The influence of various parameters on the responses of the aSPCE/SDS such as pH and concentration of the buffer, SDS concentration, and techniques parameters were investigated. Using optimised conditions (E_acc._ of −0.4 V, t_acc._ of 120 s, ΔE_A_ of 150 mV, ν of 250 mV s^−1^, and t_m_ of 10 ms), the aSPCE/SDS showed a good linear response in the concentration ranges of 5.0 × 10^−8^–2.0 × 10^−5^ for PA, 1.0 × 10^−9^–2.0 × 10^−7^ for DF, and 1.0 × 10^−8^–2.0 × 10^−7^ and 2.0 × 10^−7^–2.0 × 10^−6^ mol L^−1^ for TR. The limits of detection obtained during the simultaneous determination of PA, DF, and TR are 1.49 × 10^−8^ mol L^−1^, 2.10 × 10^−10^ mol L^−1^, and 1.71 × 10^−9^ mol L^−1^, respectively. The selectivity of the aSPCE/SDS was evaluated by examination of the impact of some inorganic and organic substances that are commonly present in environmental and biological samples on the responses of PA, DF, and TR. Finally, the differential pulse adsorptive stripping voltammetric (DPAdSV) procedure using the aSPCE/SDS was successfully applied for the determination of PA, DF, and TR in river water and serum samples as well as pharmaceuticals.

## 1. Introduction

Paracetamol (PA), also called acetaminophen, is a widely used pain reliever and antipyretic drug. However, it has no anti-inflammatory effect. Paracetamol is the main ingredient in many cold and flu medications. It is usually used to relieve headaches, toothaches, backaches, muscle aches, and other minor aches. An overdose of paracetamol may result in the accumulation of toxic metabolites that can cause acute and sometimes fatal nephro- and hepatotoxicity [1,2,3].

Diclofenac (DF) is a well-known, non-steroidal anti-inflammatory drug (NSAID) for the treatment of post-traumatic pain and pain in chronic diseases. It exhibits activities characteristic of this group of drugs, i.e., anti-inflammatory, antipyretic, analgesic, and inhibiting platelet aggregation. Despite its undoubted advantages, it can be very dangerous for living organisms. It causes an increase in blood pressure and thus strokes and worsens the functioning of the liver [2,4].

Tramadol (TR) is an opioid drug, a synthetic analog of codeine. It is a centrally acting pain reliever and is used to treat mild to severe pain. Tramadol can be used alone or in combination with NSAIDs to deal with cases associated with severe acute or chronic pain, lower back pain, and postoperative pain management. Overdosing on tramadol can cause slow or ceased breaths because this substance can accumulate in the body, causing critical levels of poisoning [5,6].

The presented pharmaceuticals may be present in the environment and have a negative impact, e.g., diclofenac affects the quality of water and is harmful to fish.

Due to the possible side effects for humans as well as the negative impact on the environment, it is extremely important to develop sensitive and accurate methods for the determination of the presented drugs in samples of biological fluids and environmental samples. There are many analytical methods such as high-performance liquid chromatography (HPLC) [7,8,9], liquid chromatography-tandem mass spectrometry (LC-MS/MS) [10,11,12], gas chromatography-mass spectrometry (GC-MS) [13,14,15], and spectrophotometry [16,17,18], which are used to determine paracetamol, diclofenac, and tramadol. However, these methods are generally costly, requiring a time-consuming sample preparation step. Compared to other analytical techniques, electrochemical methods, including voltammetry, are simple, relatively cheap, and more sensitive. In this type of procedures, apart from classic electrodes, we can use screen-printed sensors with many advantages, i.e., low cost, simplicity of construction and operation, diversification of the selection of electrode materials, portability, and ease in modification of the electrodes for various uses [19]. In the literature, we can find procedures for the determination of paracetamol alone or in the presence of various compounds [2,3,19,20,21,22,23,24,25,26,27,28,29], diclofenac [2,30,31,32], and tramadol [33] using screen-printed electrodes but there is no article showing the simultaneous determination of these three compounds.

In this work, for the first time, an electrochemically activated screen-printed carbon sensor (aSPCE) modified with an anionic surfactant (sodium dodecyl sulfate, SDS) was prepared and applied for the simultaneous determination of paracetamol, diclofenac, and tramadol. The activation can functionalize the electrode surface, increase the active surface or remove surface contamination [34,35]. Due to the adsorption of surfactants on the electrode surface, its properties change, which influences the reaction speed. Furthermore, surfactants effectively stabilize the voltammetric response by protecting the electrode surface from contamination. It has been shown that surfactants can increase the accumulation of some electroactive molecules on the electrode surface, which results in an improvement in the analytical signal and an increase in the sensitivity of the developed method [36,37,38,39,40,41,42,43,44,45,46].

## 2. Materials and Methods 

### 2.1. Apparatus

Voltammetric measurements were performed using a µAutolab analyzer (Eco Chemie, Utrecht, The Netherlands) controlled by GPES 4.9 software. All experiments were carried out in a 10 mL quartz electrochemical cell using commercially available screen-printed sensors (Ref. C150, DropSens, Llanera, Spain,), which were activated electrochemically prior to measurements. These sensors consisted of a screen-printed carbon working electrode (SPCE), a platinum screen-printed auxiliary electrode, and a silver screen-printed pseudo-reference electrode. The µAutolab analyzer (Eco Chemie, Utrecht, The Netherlands), controlled in this case by FRA 4.9 software, was also used to record the differential capacity curves and Nyquist plots by the electrochemical impedance spectroscopy (EIS) method.

Microscopic images of the SPCE surface were obtained with a high-resolution scanning electron microscope Quanta 3D FEG (FEI, Hillsboro, FL, USA). The experiments were carried out under required conditions (acceleration voltage of 5.0 kV, horizontal field width of 5.97 µm, working distance of 9.0 mm).

HPLC analyses were performed on a VWR Hitachi Elite LaChrom HPLC (Tokyo, Japan) with PDA detector using an XB-C18 reversed phase core-shell column (Kinetex, Phenomenex, Aschaffenburg, Germany) (25 cm × 4.6 mm i.d., 5 μm).

### 2.2. Reagents and Solutions

All solutions were prepared with Sigma-Aldrich or Merck reagents purchased from Merck KGaA company (Darmstadt, Germany). Appropriate amounts of paracetamol sulfate potassium salt (PA), diclofenac sodium salt (DF), and tramadol hydrochloride (TR) (Sigma-Aldrich, St. Louis, MO, USA) were dissolved in deionized water to obtain 0.01 mol L^−1^ solutions of PA and TR and 0.001 mol L^−1^ solution of DF. These solutions were diluted with deionized water as needed. A SDS (sodium dodecyl sulfate) solution was obtained by dissolving a weighed amount of Sigma-Aldrich reagent in deionized water. When selecting the base electrolyte and examining the effect of pH on the signals of the analytes, 0.1 mol L^−1^ solutions of sulfuric acid, acetic acid, and acetate buffers with pH values of 3.5 ± 0.1, 4.0 ± 0.1, 4.5 ± 0.1, 5.0 ± 0.1, 5.5 ± 0.1, and 6.0 ± 0.1 were used. DTPA (diethylenetriaminepentaacetic acid) solution was prepared in deionized water using Sigma-Aldrich reagent. The influence of interferents such as Ca(II), Mg(II), Fe(III), Ni(II), Cd(II), Pb(II), Cu(II), V(V), Mo(VI), and Cl^-^ was checked using Merck standard solutions. The effect of organic substances was checked using Sigma-Aldrich reagents: glucose, ascorbic acid, Triton X-100, and cetyltrimethylammonium bromide (CTAB). The solutions were prepared using ultrapurified water (>18 MW cm, Milli-Q system, Millipore, UK). HPLC-grade acetonitrile and trifluoroacetic acid (TFA) were from Merck. 

### 2.3. Preparation of aSPCE/SDS

The screen-printed carbon electrode surface was electrochemically activated before the measurements [34]. Activation consisted of 25 repetitive voltammetric cycles between 1.0 and −0.7 V at a scan rate of 10 mV s^−1^ in 0.1 mol L^−1^ acetate buffer of pH = 4.0 ± 0.1 containing 10 mmol L^−1^ H_2_O_2_. After activation, the sensor was rinsed with deionized water and allowed to air dry. Then, the electrode surface was modified with SDS during analysis of PA, DF, and TR by immersing the SPCE in a supporting electrolyte solution (acetate buffer of pH = 4.0 ± 0.1) containing 15 mg L^−1^ SDS. 

### 2.4. Voltammetric Analysis 

Voltammetric measurements of PA, DF, and TR in optimized conditions were carried out in a solution composed of 0.075 mol L^−1^ acetate buffer (pH of 4.0 ± 0.1), 1.0 × 10^−5^ mol L^−1^ DTPA and 15 mg L^−1^ SDS. The procedure consists of an accumulation step at a potential (E_acc._) of −0.4 V for a time (t_acc._) of 120 s. Differential pulse adsorptive stripping voltammetric (DPAdSV) curves were recorded from 0 to 2 V with an amplitude (ΔE_A_) of 150 mV, a scan rate (ν) of 250 mV s^−1^, and a modulation time (t_m_) of 10 ms. The background curve was subtracted from each voltammogram. The average values of peak current (I_p_) are shown with the standard deviation of n = 3.

### 2.5. HPLC/PDA Analysis 

For high-performance liquid chromatography photodiode array detection (HPLC/PDA), a mixture of acetonitrile and water with 0.025% of trifluoroacetic acid was used as mobile phase. The acetonitrile concentration in the eluent increased constantly from 12 to 80% during 0–30 min. The flow rate was 1.0 mL min^−1^ and temperature was set at 25°C. The injection volume was 20 µL. All samples were analyzed in triplicate, at a wavelength of 248 nm, 273 nm, and 277 nm for paracetamol, tramadol, and diclofenac, respectively.

### 2.6. Real Sample Analysis

Bystrzyca river water (Lublin, Poland), pharmaceuticals (first tablets containing PA (325 mg) and TR (37.5 mg) (Polfarmex S.A., Kutno, Poland) and second tablets containing DF (25 mg) (GSK, Brentford, UK)), and normal human serum from Merck (Darmstadt, Germany) were tested. River water samples were spiked with appropriate concentrations of the analytes and filtered through a 0.45 µm Millipore filter. Pharmaceuticals were prepared as follows. Three tablets of each drug were weighed and the average tablet weights were determined. Then, the three tablets of each drug were powdered in a mortar and the samples with corresponding average weight of one tablet were dissolved in 100 mL of deionized water. The samples were subsequently placed in an ultrasonic bath for 10 min and filtered through a 0.45 µm Millipore filter. Frozen human serum was thawed at room temperature. Then, 100 µL of the human serum sample 100 times diluted in deionized water spiked with appropriate concentrations of the analytes was transferred to a centrifugal tube, mixed with 50 µL of 7.5% (*w*/*v*) trichloroacetic acid solution (Sigma-Aldrich) for protein precipitation, centrifuged at 4000× *g* for 10 min, and filtered through a 0.45 µm Millipore filter. The collected supernatant was analyzed in triplicates by the optimized voltammetric procedure and HPLC/PDA methods.

## 3. Results and Discussion

### 3.1. Microscopic and Electrochemical Characteristic of Sensors

**Figure 1:**
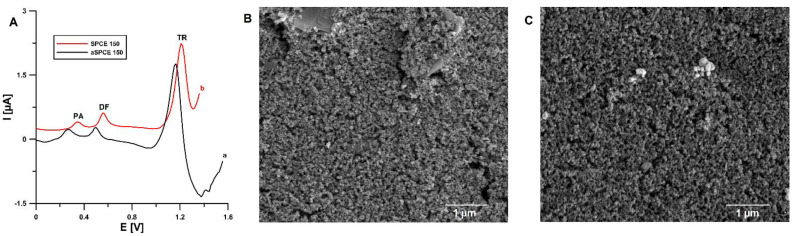
(**A**) Voltammograms of 2 × 10^−6^ mol L^−1^ PA, 1 × 10^−7^ mol L^−1^ DF, and 2 × 10^−5^ mol L^−1^ TR in 0.1 mol L^−1^ CH_3_COOH/CH_3_COONa buffer of pH 4.0 ± 0.1 recorded at the bare SPCE (a) and electrochemically activated SPCE (b). The DPAdSV parameters: E_acc._ of −0.25 V, t_acc._ of 30 s, ΔE_A_ of 50 mV, t_m_ of 50 ms, and ν of 140 mV s^−1^. SEM images with 25,000× magnification of bare SPCE (**B**) and aSPCE (**C**).

The preliminary studies (Figure 1A) showed that the application of electrochemical treatment of the SPCE surface with H_2_O_2_ [34] shifts the peak potentials of PA, DF, and TR towards less positive potential values (0.35 vs. 0.26 V for PA, 0.56 vs. 0.50 V for DF, and 1.19 vs. 1.17 V for TR) and contributes to a significant enhancement of the analytical signal of TR (1.6 vs. 2.6 µA for 2.0 × 10^−5^ mol L^−1^), with a statistically insignificant change in the peak current of PA and DF. The surface morphology of the bare SPCE and the electrochemically activated screen-printed carbon electrode (aSPCE) was examined by SEM. It was found that electrochemical activation leads to visible changes on the surface of the working electrode as the number and size of the pores increase (Figure 1B,C). This is because the organic ink constituents or contaminants introduced into the printing stage can be removed by electrochemical treatment in H_2_O_2_ [34].

**Figure 2:**
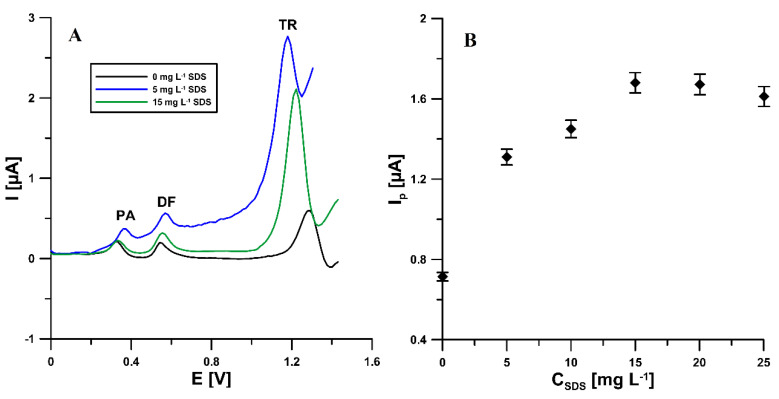
(**A**) Voltammograms of 2 × 10^−6^ mol L^−1^ PA, 1 × 10^−7^ mol L^−1^ DF, and 5.0 × 10^−6^ mol L^−1^ TR in 0.1 mol L^−1^ CH_3_COOH/CH_3_COONa buffer of pH 4.0 ± 0.1 containing 0, 5, and 15 mg L^−1^ SDS. (**B**) Influence the SDS concentration on voltammetric response of 5.0 × 10^−6^ mol L^−1^ TR. The DPAdSV parameters as in Figure 1A.

Further modification of the activated electrode surface with sodium dodecyl sulfate during the analysis of PA, DF, and TR in a supporting electrolyte solution containing SDS allows for a significant increase in the TR peak (0.74 vs. 1.7 µA for 5.0 × 10^−6^ mol L^−1^ of TR and 15 mg L^−1^ SDS), with a statistically insignificant change in the peak current of PA and DF (Figure 2A). Figure 2B shows the changes in the intensity of the TR peak current with the changing concentration of SDS. The oxidation peak current increased with the concentration of SDS, increasing from 0 to 15.0 mg L^−1^, and the response decreased when the amount of SDS further increased. The increase of the TR peak current can be explained by the electrostatic attraction between TR cations (the acidic environment) and polar groups (‘heads’) of the SDS molecules [1]. While the excess of SDS immobilizes the electrode surface, the film becomes detached and decreases the adsorption amount of tramadol. The surface morphology of the activated electrode surface modified with sodium dodecyl sulfate (aSPCE/SDS) imaged using SEM does not differ from the unmodified electrode (aSPCE) (results not shown). This is because the used concentration of SDS is below the critical micelle concentration and the surfactant in agglomerates is not visible [40]. Moreover, the influence of other surfactants (Triton X-100 and CTAB) on the analytical signals of PA, DF, and TR at the aSPCE was studied (see Section 3.5). However, the PA, DF, and TR signals decreased in the presence of CTAB and Triton-X in the supporting electrolyte. 

**Figure 3:**
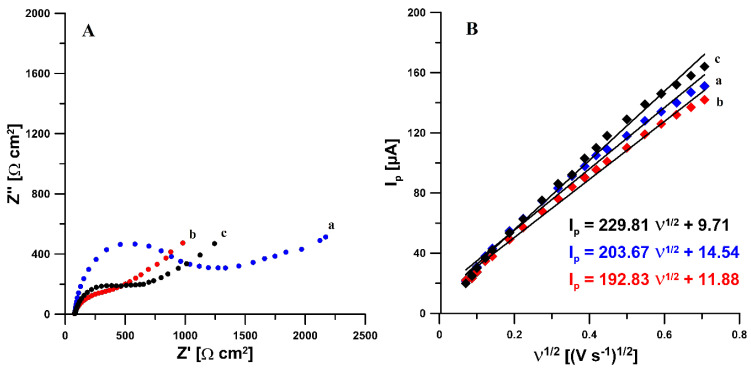
(**A**) Nyquist plots of SPCE (a), aSPCE (b), and aSPCE/SDS recorded at a potential of 0.25 V, in the frequency range from 10 kHz to 0.1 Hz, from a solution of 0.1 mol L^−1^ CH_3_COOH/CH_3_COONa buffer of pH = 4.0 ± 0.1 containing 1 × 10^−3^ mol L^−1^ PA. (**B**) Dependence between anodic peak currents and the square root of scan rates obtained in 0.1 mol L^−1^ KCl containing 5.0 mmol L^−1^ K_3_[Fe(CN)_6_] at the SPCE (a), aSPCE (b), and aSPCE/SDS (c), ν range: 5.0-500 mV s^−1^.

In order to characterize the influence of the modifications on the electrochemical properties of the sensor, measurements using electrochemical impedance spectroscopy (EIS) and cyclic voltammetry (CV) were performed. The impedance spectra (Nyquist plots) were recorded at a potential of 0.25 V in the frequency range from 10 kHz to 0.1 Hz, from a solution of 0.1 mol L^−1^ CH_3_COOH/CH_3_COONa buffer of pH = 4.0 ± 0.1 containing 1 × 10^−3^ mol L^−1^ PA, DF, or TR. As can be seen in Figure 3A obtained for the supporting electrolyte containing PA (selected example), electrochemical activation of the electrode causes a significant reduction in the value of the charge transfer resistance (R_ct_) (red curve) compared to the unactivated electrode (blue curve) (388.7 vs. 950.6 Ω cm^2^). It has also been shown that the modification of the aSPCE surface with SDS causes a slight increase in R_ct_ (black curve) compared to the activated electrode (388.7 vs. 487.6 Ω cm^2^). In addition, the active surface areas (A_s_) of the SPCE, aSPCE, and aSPCE/SDS were calculated using the Randles–Sevcik equation [47]. Figure 3B shows the relationship between anodic peak currents (I_p_) and the square root of the scan rates (v^1/2^). For the bare SPCE, aSPCE, and aSPCE/SDS, the A_s_ is equal to 0.056, 0.054, and 0.059 cm^2^, respectively. The A_s_ is almost the same for all studied electrodes. 

To sum up, the SPCE morphology surface changed greatly after electrochemical treatment with H_2_O_2_. This contributes to lowering the charge transfer resistance of the electrode. The SDS modification slightly increases the charge transfer resistance of the activated electrode but does not block the electrode surface, whereas the active surface areas for the bare SPCE, aSPCE, and aSPCE/SDS are almost the same. 

### 3.2. Influence of pH

**Figure 4:**
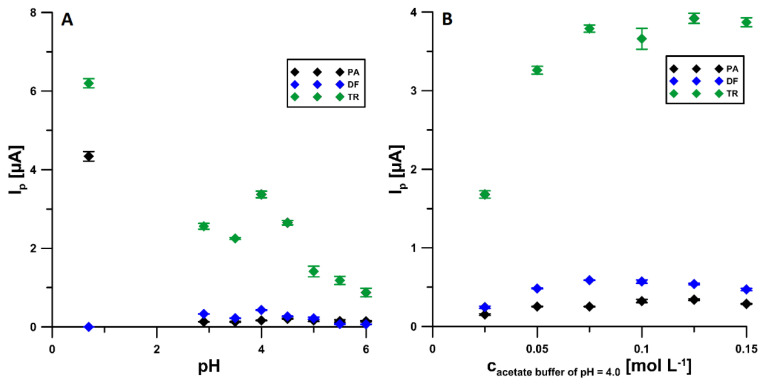
Effect of pH (**A**) and the concentration of the CH_3_COOH/CH_3_COONa buffer solution with a pH of 4.0 ± 0.1 (**B**) on the signals of 2 × 10^−6^ mol L^−1^ PA, 1 × 10^−7^ mol L^−1^ DF, and 2 × 10^−5^ mol L^−1^ TR. The DPAdSV parameters as in Figure 1A.

The effect of the type and pH of the supporting electrolyte on the signals of 2 × 10^−6^ mol L^−1^ PA, 1 × 10^−7^ mol L^−1^ DF, and 2 × 10^−5^ mol L^−1^ TR was investigated for 0.1 mol L^−1^ of H_2_SO_4_, CH_3_COOH solutions, and CH_3_COOH/CH_3_COONa buffers with pH of 3.5 ± 0.1, 4.0 ± 0.1, 4.5 ± 0.1, 5.0 ± 0.1, 5.5 ± 0.1, and 6.0 ± 0.1. The corresponding data are presented in Figure 4A. As can be seen for the simultaneous determination of PA and TR, a sulfuric acid solution should be used as the supporting electrolyte. However, in the case of the simultaneous determination of PA, DF, and TR, the acetate buffer at pH of 4.0 ± 0.1 is the best choice considering the peak currents. In addition, the influence of the concentration of the selected supporting electrolyte on the voltammetric response of the analytes was also checked (Figure 4B) and it was shown that the highest peak current values were obtained for 0.075 mol L^−1^ CH_3_COOH/CH_3_COONa buffer of pH of 4.0 ± 0.1, hence it was used for further research.

### 3.3. Adsorption Studies

**Figure 5:**
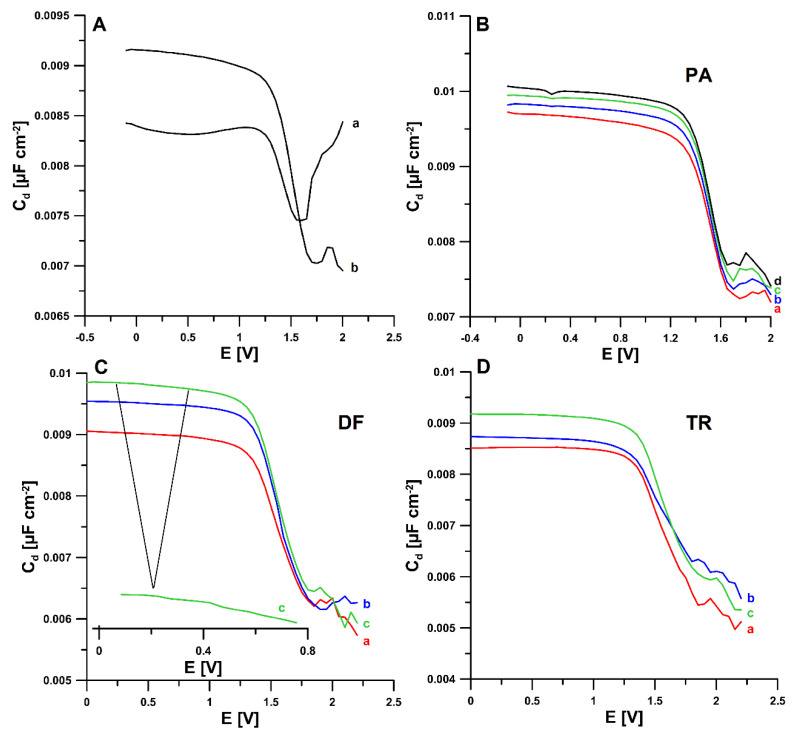
The differential capacity-potential curves of the double layer interface aSPCE/acetate buffer of pH = 4.0 ± 0.1 in the presence of: (**A**) 0 (a) and 15 (b) mg L^−1^ SDS, (**B**) 15 mg L^−1^ SDS and (a) 2 × 10^−7^, (b) 2 × 10^−6^, (c) 2 × 10^−5^, (d) 2 × 10^−4^ mol L^−1^ PA, (**C**) 15 mg L^−1^ SDS and (a) 2 × 10^−7^, (b) 2 × 10^−6^, (c) 2 × 10^−5^ mol L^−1^ DF, (**D**) 15 mg L^−1^ SDS and (a) 2 × 10^−7^, (b) 2 × 10^−6^, (c) 2 × 10^−5^ mol L^−1^ TR.

The information on the electrochemical response of PA, DF, and TR on the aSPCE/SDS was obtained from the analysis of differential capacity curves. For each analyte, measurements were made at a frequency of 200 Hz in the potential range of −0.1 to 2 V. Based on the obtained results, it can be concluded that the SDS used as a modifier adsorbs onto the aSPCE surface, which is evidenced by the difference in the curves of the double layer interface aSPCE/acetate buffer of pH = 4.0 in the absence and presence of 15 mg L^−1^ SDS (Figure 5A). No adsorption peaks of any of the analytes occurred in the potential range used. However, in the presence of PA, a desorption peak can be seen at a potential of 0.25 V, the height of which increases with the increasing concentration of PA in the solution (Figure 5B). This proves the strong adsorption of PA. In the case of adding DF, for a concentration of 2 × 10^−6^ mol L^−1^ and higher, two very small desorption peaks (0.3 and 0.45 V) can be noticed, which may indicate slight adsorption of this analyte on the aSPCE/SDS surface (Figure 5C). Figure 5D shows the differential capacity curves recorded for increasing TR concentrations. No peak was observed here, which makes it possible to conclude that TR existing in the cationic form reaches the electrode by diffusion and becomes electrostatically attracted by the surface adsorbed SDS anions.

### 3.4. Optimization of Procedure Parameters 

**Figure 6:**
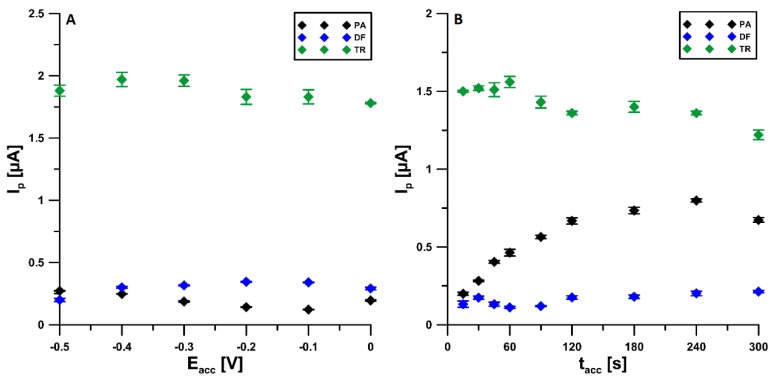
Influence of E_acc._ (**A**) and t_acc._ (**B**) on voltammetric response of 2 × 10^−6^ mol L^−1^ PA, 1 × 10^−7^ mol L^−1^ DF, and 1 × 10^−5^ mol L^−1^TR. The DPAdSV parameters: ΔE_A_ of 50 mV, t_m_ of 50 ms, and ν of 140 mV s^−1^.

In order to find the most optimal conditions for the analysis of PA, DF, and TR at the aSPCE/SDS, the effect of parameters such as the accumulation potential (E_acc._) and time (t_acc._), amplitude (ΔE_A_), scan rate (ν), and modulation time (t_m_) on the peak currents was investigated. The effect of E_acc_ was tested in the range from 0 to −0.5 V with the t_acc._ of 30 s. The highest signals were obtained at a potential of −0.4 V (Figure 6A). Then, for the selected value of the potential, the effect of t_acc._ in the range of 15–300 s was investigated. The t_acc._ of 120 s was chosen (Figure 6B), but the accumulation stage can be extended to obtain lower detection limits.

**Figure 7:**
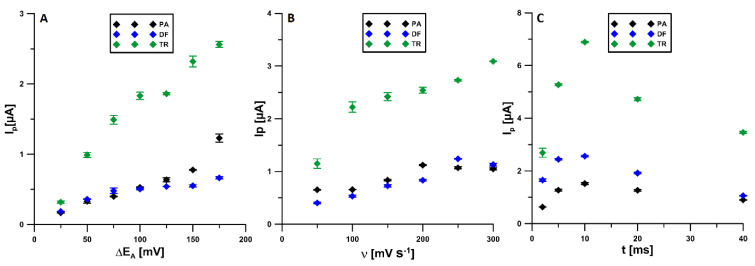
Influence of ΔE_A_ (**A**), ν (**B**), and t_m_ (**C**) on voltammetric response of 2 × 10^−6^ mol L^−1^PA, 1 × 10^−7^ mol L^−1^ DF, and 1 × 10^−5^ mol L^−1^ TR. The DPAdV parameters: E_acc._ of −0.4 V and t_acc._ of 120 s.

The ΔE_A_ varied from 25 to 175 mV. For further experiments, the value of 150 mV was selected (Figure 7A). Then, the effect of ν in the range of 50–300 mV s^−1^ was checked. It was found that the highest PA and DF signals were recorded for ν equal to 250 mV s^−1^ and this value was considered as the most optimal. For ν equal to 300 mV s^−1^, the TR peak was higher, but the PA and DF signals decreased (Figure 7B). The last analyzed parameter was t_m_ checked in the range of 2 to 40 ms. The highest signals of all three tested compounds were recorded for the t_m_ of 10 ms (Figure 7C).

### 3.5. Selectivity 

In the optimized conditions, the influence of potential interferents on the determination of PA, DF, and TRA was investigated. The tolerance limit was defined as the concentration, which gave an error of ≤ 10% in the determination of 5 × 10^−6^ mol L^−1^ PA, 2 × 10^−7^ mol L^−1^ DF, and 2 × 10^−6^ mol L^−1^ TR. It needs to be highlighted that the addition of 1.0 × 10^−5^ mol L^−1^ DTPA to the supporting electrolyte solution was applied. This was to minimize the interference from metal ions due to their complexation with DTPA. It was noticed that an excess of glucose (up to 100-fold), ascorbic acid (up to 10-fold), Fe(III) (up to 10-fold), Ca(II) (up to 40-fold), Cu(II) (up to 2-fold), Mg(II) (up to 100-fold), Cd(II) (up to 10-fold), Pb(II) (up to 1000-fold), Ni(II) ions (up to 10-fold), Mo(VI) (up to 200-fold), and Cl(-I) (up to 20-fold) had negligible effects on the assay of PA. It was observed that an excess of glucose (up to 2500-fold), ascorbic acid (up to 100-fold), Fe(III) (up to 100-fold), Ca(II) (up to 100-fold), Cu(II) (up to 25-fold), Mg(II) (up to 500-fold), Cd(II) (up to 10-fold), Pb(II) (up to 1000-fold), Ni(II) (up to 25-fold), Mo(VI) (up to 500-fold), and Cl(-I) (up to 2500-fold ex) had negligible effects on the assay of DF. Moreover, an excess of glucose (up to 2500-fold), ascorbic acid (up to 5-fold), Fe(III) (up to 100-fold), Ca(II) (up to 500-fold), Cu(II) (up to 10-fold), Mg(II) (up to 250-fold), Cd(II) (up to 25-fold), Pb(II) (up to 1000-fold), Ni(II) (up to 25-fold), Mo(VI) (up to 50-fold), and Cl(-I) (up to 25-fold) had negligible effects on the assay of TR. 

**Figure 8:**
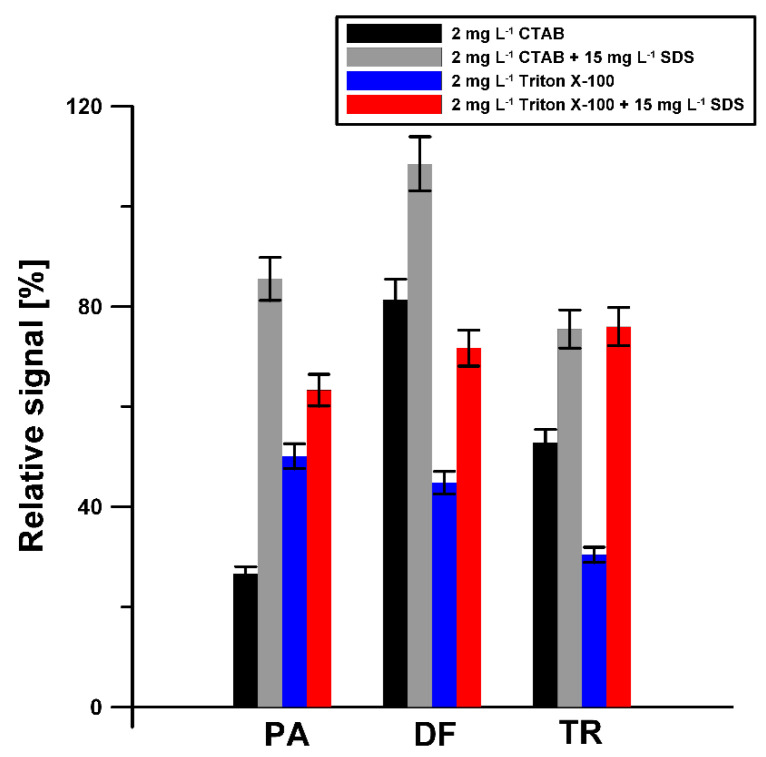
Influence of 2 ppm Triton X-100 and CTAB on voltammetric response of 5 × 10^−6^ mol L^−1^ PA, 2 × 10^−7^ mol L^−1^ DF, and 2 × 10^−6^ mol L^−1^ TR in the absence and presence of 15 mg L^−1^ SDS.

Due to the fact that natural waters contain surfactants with a surface active effect comparable to the effect of 0.2 to 2 mg L^−1^ of Triton X-100 [48], the influence of 2 mg L^−1^ of Triton X-100 on the voltammetric response of 5 × 10^−6^ mol L^−1^ PA, 2 × 10^−7^ mol L^−1^ DF, and 2 × 10^−6^ mol L^−1^ TR was investigated. Moreover, the influence of cationic surfactant (CTAB) was studied. As can be seen in Figure 8, the adsorption of SDS on the aSPCE surface contributes to minimizing the effect of the surfactants (Triton X-100 and CTAB) on the analytical signal of all analyzed substances. In the presence of 2 ppm of Triton X -100, 2 ppm of CTAB, and 15 mg L^−1^ SDS in the supporting electrolyte, the signals do not fall below 60% of their original values and are well formed and easy to measure.

### 3.6. Analytical Characteristic

**Figure 9:**
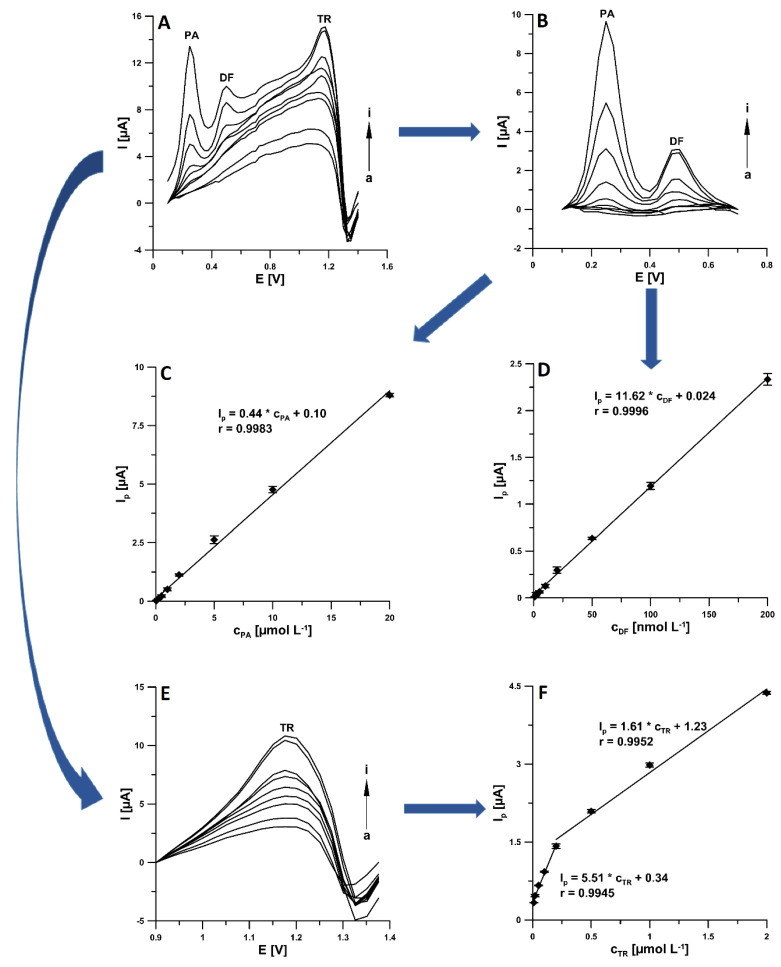
(**A**) Voltammograms obtained at the aSPCE/SDS in 0.075 mol L^−1^ CH_3_COOH/CH_3_COONa buffer solution of pH 4.0 ± 0.1 containing increasing concentrations of PA (a–i, 0.05, 0.1, 0.2, 0.5, 1, 2, 5, 10, 20 µmol L^−1^), DF (a–i, 0.001, 0.002, 0.005, 0.01, 0.02, 0.05, 0.1, 0.2, 0.2 µmol L^−1^), TR (a–i, 0.01, 0.02, 0.05, 0.1, 0.2, 0.5, 1, 2, 2 µmol L^−1^). (**B**) Segment of voltammograms (A) in the potential range of 0.1–0.7 V. Linear range of the calibration curve of PA (**C**) and DF (**D**,**E**) Segment of voltammograms (A) in the potential range of 0.9–1.4 V. (**F**) Linear ranges of the calibration curve of TR. The DPAdSV parameters: E_acc._ of −0.4 V, t_acc._ of 120 s, ΔE_A_ of 50 mV, t_m_ of 10 ms, and ν of 250 mV s^−1^.

Under the optimized conditions, the ability of the aSPCE/SDS for individual and simultaneous determination of PA, DF, and TR was studied. The results are summarized in Table 1. Figure 9 shows the voltammograms and linear ranges of the calibration plots obtained during simultaneous determination of PA, DF, and PA. The limits of detection (LOD) and quantification (LOQ) obtained during simultaneous determination of PA, DF, and TR are 14.87, 0.21, and 1.71 nmol L^−1^, and 49.56, 0.69, and 5.69 nmol L^−1^, respectively, according to the definitions of LOD = 3SD_a_/b and LOQ = 10SD_a_/b (SD_a_—standard deviation of intercept (n = 3); b—slope of calibration curve) [49]. Table 2 shows the comparison techniques used for the determination of PA, DF, and TR. It should be clearly emphasized that the proposed voltammetric procedure using the aSPCE/SDS mostly allows a significantly lower LOD to be obtained than those obtained for other techniques [7,8,9,10,11,12,14,16,17,18,47]. In the case of the article [9], the calculated LOD of TR is lower (5.33 × 10^−10^ vs. 1.71 × 10^−9^ mol L^−1^), but the first concertation of TR from the calibration graph is higher than the one obtained at the aSPCE/SDS (1.67 × 10^−8^ vs. 1.0 × 10^−8^ mol L^−1^). On the other hand, in the article [15], the LOD is equal to the first concertation of TR, which is incorrect. Moreover, both techniques [9,15] require more expensive equipment, the procedures are more laborious, and more reagents are used. Furthermore, it should be emphasized that this is the first electrochemical sensor for simultaneous determination of PA, DF, and TR.

Additionally, the precision was verified for the determination of 5 × 10^−6^ mol L^−1^ PA, 2 × 10^−7^ mol L^−1^ DF, and 2 × 10^−7^ mol L^−1^ TR with ten replicates. The results were 2.7, 1.2, and 1.8%, respectively, indicating the satisfactory repeatability of the signals at the aSPCE/SDS. The reproducibility was assessed based on voltammograms registered in the solution containing 1 × 10^−6^ mol L^−1^ PA, 1 × 10^−8^ mol L^−1^ DF, and 2 × 10^−7^ mol L^−1^ TR at three freshly prepared electrodes. The RSD was calculated as 2.5, 3.1, and 3.5% (n = 6), respectively, confirming the acceptable reproducibility of the aSPCE/SDS.

### 3.7. Sample Analysis

In order to confirm the usefulness of the developed voltammetric procedure at the aSPCE/SDS, the simultaneous determination of PA, DF, and TR was carried out in Bystrzyca river water samples and human serum samples, the results of which are presented in Table 3. The recovery values obtained by DPV were between 97.0 and 102.0%, which corresponds to a satisfactory degree of accuracy of the method. The HPLC/PDA was used as a comparative method for the determination of PA, DF, and TR in river water and human serum samples. However, the concentrations of PA, DF, and TR were below the detection limits of the chromatographic method. The calculated LOD by HPLC/PDA for PA, DF, and TR was 2.4 × 10^−7^, 5.2 × 10^−7^, and 2.7 × 10^−7^ mol L^−1^, respectively. Moreover, the contents of the analytes, PA and TR in the pharmaceutical tablets 1 and DF in the pharmaceutical tablets 2, were determined. The results are summarized in Table 4. The obtained values are consistent with the values declared by the manufacturer. The calculated relative errors (0–2.1%) indicated that there were no important matrix interferences for the pharmaceuticals analyzed by the proposed DPAdSV procedure at the SPCE/SDS.

## 4. Conclusions

In summary, in this study, an electrochemically activated screen-printed carbon electrode modified with sodium dodecyl sulfate (aSPCE/SDS) was introduced for the first time for the simultaneous analysis of paracetamol (PA), diclofenac (DF), and tramadol (TR). The electrochemical activation of the SPCE surface using CV in acetate buffer of pH = 4.0 ± 0.1 containing H_2_O_2_ significantly changes the electrode surface morphology and reduces the charge transfer resistance. The modification with SDS allows for the enhancement of the TR signal, while not negatively affecting the PA and DF signals, and greatly minimizes the influence of surfactants (Triton X-100 and CTAB) on the analytical signal of all analyzed substances. The DPAdSV procedure with the aSPCE/SDS allows for selective determination of low PA, DF, and TR concentrations. The LODs and LOQs obtained during simultaneous determination of PA, DF, and TR are 14.87 and 49.56 nmol L^−1^, 0.21 and 0.69 nmol L^−1^, and 1.71 and 5.69 nmol L^−1^, respectively. The developed sensor was successfully used to determine PA, DF, and TR in river water and human serum samples as well as in pharmaceutical preparations. The concentrations of PA, DF, and TR determined by DPAdSV method in river water and human serum samples were below the detection limits of the chromatographic method (HPLC/PDA). The obtained results show that the procedure can be used as a quick, simple, and cheap alternative to other methods. Moreover, it should be highlighted that the further advantage of the aSPCE/SDS sensor is it portability, which is very promising for quick field analysis.

## Figures and Tables

**Table 1:** Analytical parameters obtained for individual and simultaneous determination of PA, DF, and TR at the aSPCE/SDS.

Parameter	PA	DF	TR	PA, DF, and TR
Linear range (nmol L^−1^)	50–100000	1–200	10–200 200–2000	50–20,000 (PA)
				1–200 (DF)
				10–200 (TR)
				200–2000 (TR)
Calibration graph equation I_p_ (µA) c_PA_ (µmol L^−1^) c_DF_ (nmol L^−1^) c_TR_ (µmol L^−1^)	I_p_ = 0.30c_PA_ + 0.77	I_p_ =14.27c_DF_ + 0.063	I_p_ = 5.95c_TR_ + 0.33 I_p_ = 1.63c_TR_ + 1.31	I_p_ = 0.44c_PA_ + 0.10
				I_p_ = 11.62c_DF_ + 0.024
				I_p_ = 5.51c_TR_ + 0.34
				I_p_ = 1.61 c_TR_ + 1.23
Correlation coefficient (r)	0.9947	0.9967	0.9940 0.9947	0.9983 (PA)
				0.9996 (DF)
				0.9945 (TR)
				0.9952 (TR)
LOD (nmol L^−1^)	12.93	0.12	2.47	14.87 (PA)
				0.21(DF)
				1.71 (TR)
LOQ (nmol L^−1^)	43.09	0.39	8.24	49.56 (PA)
				0.69 (DF)
				5.69 (TR)

**Table 2:** Comparison of techniques for analysis of PA, DF, and TR.

Method	Analyte	Linear Range (mol L^−1^)	LOD (mol L^−1^)	Application	Ref.
HPLC	PA	5.74 × 10^−4^–9.93 × 10^−4^	1.50 × 10^−5^	Pharmaceutical formulations	[7]
HPLC	DF	3.14 × 10^−9^–2.30 × 10^−6^ 1.26 × 10^−8^–1.97 × 10^−6^	3.77 × 10^−10^ 1.57 × 10^−9^	Hospital wastewater, human serum	[8]
HPLC	TR	1.67 × 10^−8^–1.0 × 10^−6^	5.33 × 10^−10^	Urine, plasma	[9]
LC-MS/MS	PA	8.28 × 10^−7^–3.31 × 10^−4^	2.21 × 10^−8^	Human plasma	[10]
LC-MS/MS	DF	1.57 × 10^−8^–3.14 × 10^−5^	6.29 × 10^−9^	Cow plasma	[11]
LC-MS/MS	TR	6.67 × 10^−8^–8.33 × 10^−7^	8.66 × 10^−9^	Pharmaceutical formulations	[12]
GC-MS	PA	4.97 × 10^−4^–3.31 × 10^−3^	1.32 × 10^−4^	Pharmaceutical formulations	[47]
GC-MS	DF	7.86 × 10^−10^–1.57 × 10^−7^	3.93 × 10^−10^	Human plasma	[14]
GC-MS	TR	1.50 × 10^−9^–1.0 × 10^−6^ 1.83 × 10^−8^–8.33 × 10^−7^ 8.33 × 10^−9^–1.0 × 10^−6^	1.50 × 10^−9^8.33 × 10^−9^2.67 × 10^−9^	Plasma, urine, saliva	[15]
Spectrophotometry	PA	0–9.94 × 10^−4^	-	Pharmaceutical formulations	[16]
Spectrophotometry	DF	1.57 × 10^−5^–2.52 × 10^−4^	-	Pharmaceutical formulations	[17]
Spectrophotometry	TR	5.67 × 10^−6^–1.43 × 10^−5^	-	Pharmaceutical formulations	[18]
DPAdSV	PA DF	5.0 × 10^−8^–2.0 × 10^−5^ 1.0 × 10^−9^–2.0 × 10^−7^	1.49 × 10^−8^2.10 × 10^−10^	River water, human serum,	This work
	TR	1.0 × 10^−8^–2.0 × 10^−7^ 2.0 × 10^−7^–2.0 × 10^−6^	1.71 × 10^−9^	pharmaceutical formulations	

**Table 3:** The results of simultaneous PA, DF, and TR determination in river water and human serum samples.

**Sample**	**PA Concentration (nmol L^−1^) ± SD (n = 3)**			Recovery * (%)
	**Added**	**Found DPAdSV**	**Found HPLC/PDA**	
Bystrzyca river	0 200	<LOD 191 ± 9.0	<LOD <LOD	- 97.0
Human serum	0	<LOD	<LOD	-
	200	204 ± 1.2	<LOD	102.0
**Sample**	**DF concentration (nmol L^−1^) ± SD (n = 3)**			Recovery * (%)
	**Added**	**Found DPAdSV**	**Found HPLC/PDA**	
Bystrzyca river	0 20	<LOD 20.1 ± 0.2	<LOD <LOD	- 100.5
Human serum	0	<LOD	<LOD	-
	20	19.7 ± 1.0	<LOD	98.5
**Sample**	**TR concentration (nmol L^−1^0 ± SD (n = 3)**			Recovery * (%)
	**Added**	**Found DPAdSV**	**Found HPLC/PDA**	
Bystrzyca river	0 20	<LOD 20.4 ± 0.6	<LOD <LOD	- 102.0
Human serum	0	<LOD	<LOD	-
	20	20.0 ± 0.4	<LOD	100.0

* Recovery (%) = (Found DPAdSV × 100)/Added.

**Table 4:** The results obtained during the determination of PA, DF, and TR in pharmaceutical formulations at the aSPCE/SDS.

Tablets	Compound	Label Value (mg)	Determined DPAdSV (mg) ± SD (n = 3)	Relative Error * (%)
1	PA	325.0	321.3 ± 3.8	1.1
	TR	37.5	38.3 ± 2.1	2.1
2	DF	25.0	25.0 ± 0.64	0.0

* Relative error (%) = (|DPAdSV value − label value|/label value) × 100.

## Data Availability

Not applicable.
